# Continuous Local Infiltration Analgesia for Pain Control After Total Knee Arthroplasty: A Meta-analysis of Randomized Controlled Trials

**DOI:** 10.1097/MD.0000000000002005

**Published:** 2015-11-13

**Authors:** Xiao-Lei Sun, Zhi-Hu Zhao, Jian-Xiong Ma, Feng-Bo Li, Yan-Jun Li, Xin-Min Meng, Xin-Long Ma

**Affiliations:** From the Department of Orthopaedics, Tianjin Hospital (X-LS, Z-HZ, J-XM, F-BL, Y-JL, X-MM, X-LM); and Graduate School of Tianjin Medical University, Tianjin, China (Z-HZ).

## Abstract

A total knee arthroplasty (TKA) has always been associated with moderate to severe pain. As more research is conducted on the use of continuous local infiltration analgesia (CLIA) to manage pain after a TKA, it is necessary to reassess the efficacy and safety of the TKA method. The purpose of this systematic review and meta-analysis of randomized controlled trials was to evaluate the efficacy and safety of pain control of CLIA versus placebo after a TKA.

In January 2015, a systematic computer-based search was conducted in the Medline, Embase, PubMed, CENTRAL (Cochrane Controlled Trials Register), Web of Science, Google database, and Chinese Wanfang databases. This systematic review and meta-analysis were performed according to the Preferred Reporting Items for Systematic Reviews and Meta-analyses statement criteria. The primary endpoint was the visual analog scale score after a TKA with rest or mobilization at 24, 48, and 72 hours, which represents the effect of pain control after TKA. The complications of infection, nausea, and whether it prolonged wound drainage were also compiled to assess the safety of CLIA. RevMan 5.30 software was used for the meta-analysis. After testing for publication bias and heterogeneity across studies, data were aggregated for random-effects modeling when necessary.

Ten studies involving 735 patients met the inclusion criteria. The meta-analysis revealed that continuous infusion analgesia provided better pain control with rest at 24 hours (mean difference [MD] −12.54, 95% confidence interval [CI] −16.63 to 8.45), and with mobilization at 24 hours (MD −18.27, 95% CI −27.52 to 9.02) and 48 hours (MD −14.19, 95% CI −21.46 to 6.93). There was no significant difference with respect to the visual analog scale score at 48 hours (MD −6.15, 95% CI −13.51 to 1.22, *P* = 0.10) and 72 hours (MD −3.63, 95% CI −10.43 to 3.16, *P* = 0.29) with rest and at 72 hours with mobilization (MD −4.25, 95% CI −16.27 to 7.77, *P* = 0.49). However, CLIA increased the rate of infection (relative risk [RR] 3.16, 95% CI 1.18–8.50, *P* = 0.02) and the rate of nausea or vomiting (RR 0.60, 95% CI 0.37–0.96, *P* = 0.03). There were no significant differences in the length of hospital stay (MD −0.34, 95% CI −1.09 to 0.42, *P* = 0.38), deep venous thrombosis (RR 1.02, 95% CI 0.30 to 1.41, *P* = 0.99), or duration of surgery (MD 1.20, 95% CI −4.59 to 6.98, *P* = 0.69).

On the basis of the current meta-analysis, CLIA was more efficacious for reducing postoperative pain than the placebo at 24 hours with rest and at 24 and 48 hours with mobilization, but it increased the risk of infection. However, CLIA did not prolong the length of hospital stay or the duration of surgery. There was also a higher heterogeneity of different analgesic drugs mixed and a high risk of selection bias in this analysis; therefore, more high-quality randomized controlled trials with standardized CLIA are necessary for proper comparisons of this technique with other methods.

## INTRODUCTION

Total knee arthroplasty (TKA) is one of the most effective surgeries for improving the quality of life of end-stage patients with osteoarthritis or rheumatic arthritis (RA) of the knee; however, TKA has always been associated with a relatively intense amount of pain that is difficult to manage.^1^ It has been reported that approximately 60% of patients have severe pain and 30% of patients have moderate pain after TKA.^2^ In addition, the pain after TKA is more intense during mobilization; consequently, patients tend to prefer the comfort of a hospital bed, which prolongs the length of hospital stay (LOS), increases medical expenses, and increases the risk of a deep venous thrombosis (DVT) or a pulmonary embolism (PE).^3,4^

A variety of modalities have been applied to control the postoperative pain after a TKA; these methods include single-injection local infiltration analgesia (SLIA), oral opiates, and epidural infusions.^5–7^ SLIA has been identified to provide good pain control in comparison with systemic opioids, and it reduces opioid consumption after TKA.^8–10^ However, the duration of the analgesic effect of SLIA is 8 to 12 hours and potentially as long as 48 hours. Systemic analgesics, such as opioids, might increase the analgesic-related complications and the duration of a postoperative ileus.^11^ Attaining postoperative pain relief after a TKA and reducing morphine consumption are crucial to improve the quality of life of TKA patients and to shorten hospitalization stays.

Continuous local infiltration analgesia (CLIA) has been shown to have the same effect as SLIA on pain management after TKA while also prolonging the effect of pain control via reliable elastomeric pumps and multihole catheters. The best advantage of CLIA is that it allows early physiotherapy because it has no motor blockade impacting the strength of the muscles. Several randomized controlled trials (RCTs) have estimated the efficacy of utilizing a local anesthetic infusion pump in patients undergoing CLIA; however, the effects and the wound infection complications have been inconclusive. To our surprise, Ali et al^12^ reported that CLIA has no effect on the visual analog scale (VAS) score, but increases the risk of infection. Therefore, the purpose of this meta-analysis is to assess the efficacy and safety of CLIA in the management of pain after a TKA by examining the VAS score with rest or mobilization at 24, 48, and 72 hours after surgery, and the impact of CLIA on surgery time and the risk of infection.

## MATERIALS AND METHODS

### Search Strategy

The following electronic databases were searched for relevant academic trials comparing CLIA to placebo for the management of pain after a TKA from inception to January 2015: Medline, Embase, PubMed, CENTRAL (Cochrane Controlled Trials Register), Web of Science, Chinese Wanfang, and Google. The key words and medical subject heading (MESH) terms included the following: continuous local infiltration analgesia, local anesthesia, local anesthetic, continuous infusion, pump, pain control, total knee arthroplasty, total knee replacement, TKA, and TKR. Because a specific definition of CLIA has not reached a consensus, we defined CLIA as continuously applied analgesia drugs via a catheter, with a pump or bolus injection after a TKA. These key words and the corresponding MESH terms were combined with the Boolean operators “AND” and “OR.” Furthermore, the reference lists of all of the full-text literature were reviewed to identify any initially omitted studies, and no restriction was made on the language of the publication. Two reviewers (Z-HZ and X-LS) independently searched the databases and filtered the relevant literature. Conflicts were resolved by the third reviewer (X-LM). Next, the full articles were downloaded to screen whether articles fit the inclusion and exclusion criteria. Because this is a meta-analysis, so there was no ethics committee or institutional review board to approve the study.

### Inclusion Criteria and Study Selection

Inclusion criteria were as follows: RCTs; patients who underwent a primary TKA; interventions, including CLIA with a control (placebo or nothing); and reported outcomes, including postoperative VAS pain with rest or mobilization at 24 and 48 hours, the incidence of infection, surgery time, and the LOS. The article should include at least one of the outcomes mentioned above. We excluded studies on cadaver or artificial models. We also excluded non-RCTs, letters, comments, editorials, practice guidelines, and other studies with insufficient data.

### Data Abstraction and Quality Assessment

After duplicates were excluded, 2 reviewers independently read the titles and abstracts of the searched literature. Most of the articles were excluded based on the topic of the article provided in the respective title or abstract, and disagreements about whether or not an article was included were resolved by discussion or by a senior reviewer. Postoperative pain intensity was measured by a 100-point VAS. The 10-point VAS score was converted to a 100-point VAS score. Data in other forms (ie, median, interquartile range, and mean ± 95% confidence interval [CI]) were converted to mean ± SD according to the Cochrane Handbook.^13^ If the data were not reported numerically, we extracted them using the “GetData Graph Digitizer” software from the published figures.

The following data were extracted and recorded in a sheet: demographic data about the patients in the literature, the author's name, the publication date, the sample size, the location of the study, and the ratio of males to females; the method of surgery and anesthesia; the technique of infusion, the injection location, and the dose of injection drugs; and the VAS score with rest or mobilization at 24 and 48 hours, the rates of infection, and the LOS. Two reviewers (Z-HZ and X-LS) scanned the quality of the eligible studies independently. The quality of the RCT studies was judged using the Jadad 5-point scale. Discrepancies were resolved by consensus after discussion, and a third reviewer participated in the debate to determine the final outcome if necessary. The risk of bias for each RCT was evaluated using the Cochrane Collaboration Risk of Bias Tool.

### Statistical Analysis

Continuous outcomes, such as the VAS score with rest or mobilization at 24, 48, and 72 hours; the LOS; and the duration of surgery were expressed as the mean difference (MD) with the respective 95% CIs. Discontinuous outcomes (the rate of infection, DVT, prolonged wound drainage, and nausea/vomiting) were expressed as the relative risk (RR) with 95% CIs. Statistical significance was set at *P* < 0.05 to summarize findings across the trials. RevMan 5.30 software (The Cochrane Collaboration, Oxford, UK) was used for the meta-analysis. Statistical heterogeneity was tested using the chi-square test and *I*^2^ statistic. A chi-square test *I*^2^ >50% was considered suggestive of statistical heterogeneity. When there was no statistical evidence of heterogeneity, a fixed-effect model was adopted; otherwise, a random-effect model was chosen.

## RESULTS

### Search Results

In the initial search, we identified 682 potentially relevant studies, of which 176 duplicates were removed by Endnote Software. According to the inclusion criteria, 235 studies were excluded after reading the titles and abstracts. Finally, we included 10 clinical trials with 735 patients in the meta-analysis.^14–23^ The characteristics of the included studies are shown in Table 1 . In the included studies, a total of 587 TKAs were performed, and the numbers of studies using continuous infusion analgesia and saline were 295 and 292, respectively. Only 1 article was published in 2005; the remaining articles were published in 2009. All of the participants in the 5 studies were older, ranging in age from 65 to 72 years. There were 273 male patients and 314 female patients. The methods that maintained continuous infusion included a pain pump and an elastomeric balloon at speeds ranging from 2 to 5 mL/h. The risk of bias for the included studies and GRADE of evidence are shown in Table 2.

**TABLE 1:**
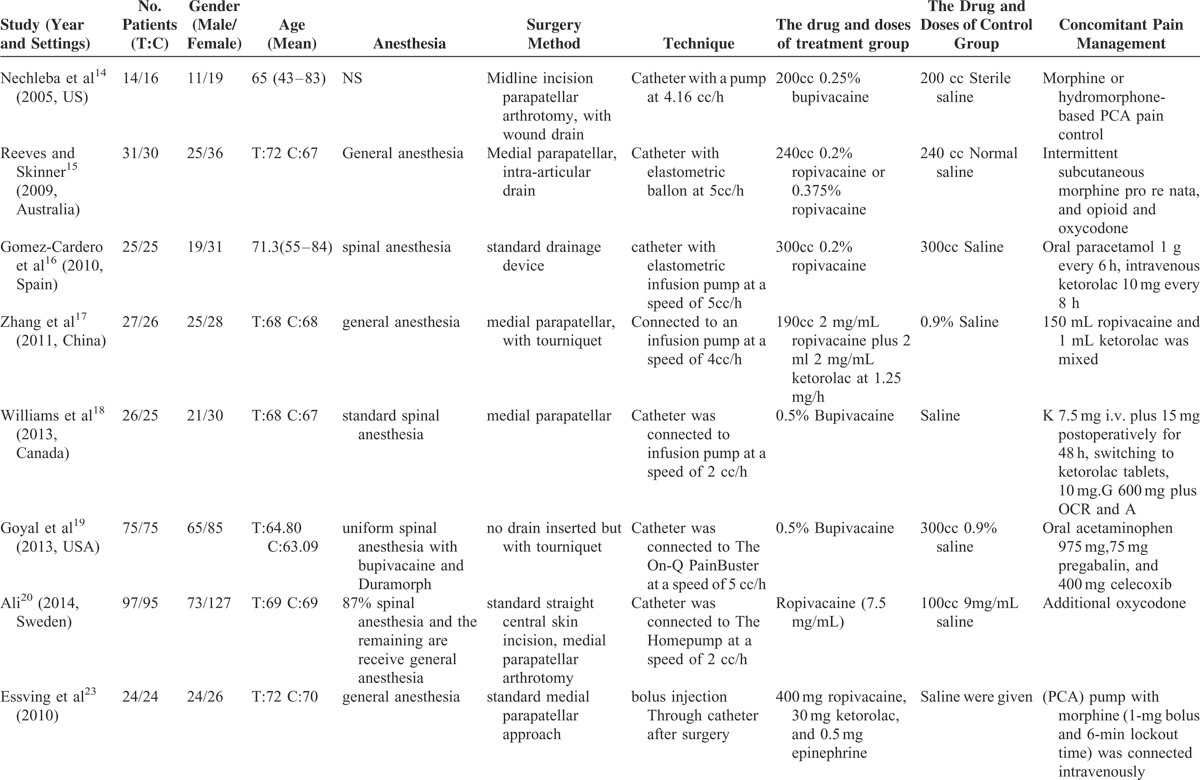
Characteristics of Included Studies

**TABLE 1 (Continued):**
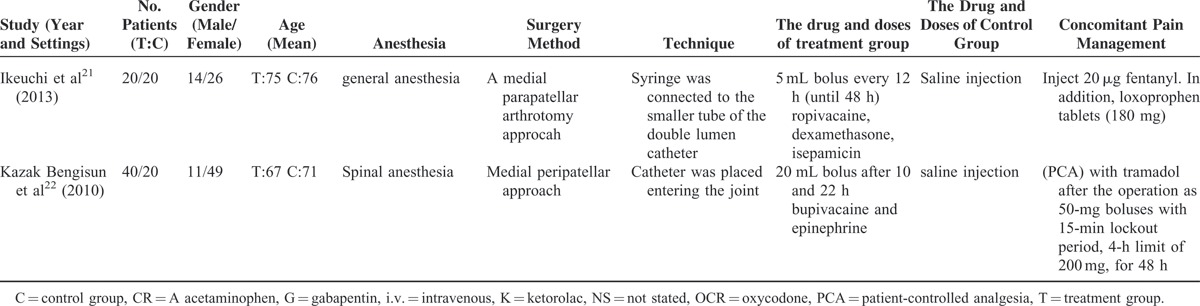
Characteristics of Included Studies

**TABLE 2:**
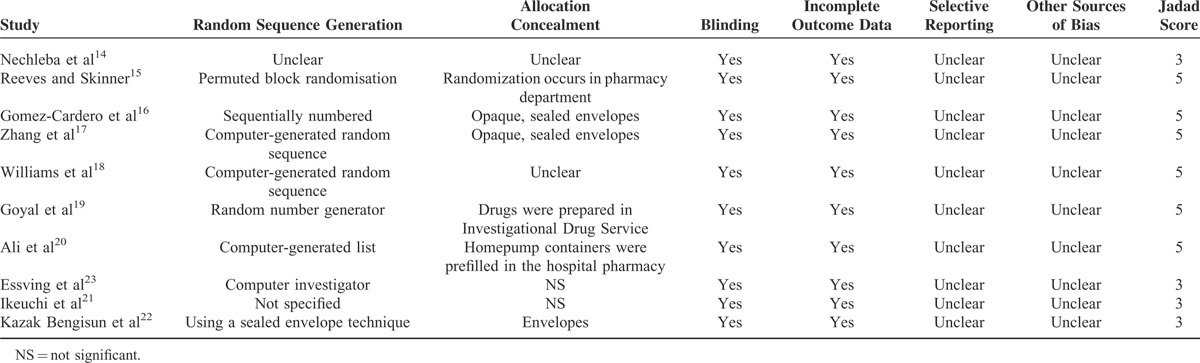
The Risk Bias of the Included Studies

### Result of Meta-analysis

#### VAS Score With Rest

**FIGURE 1:**
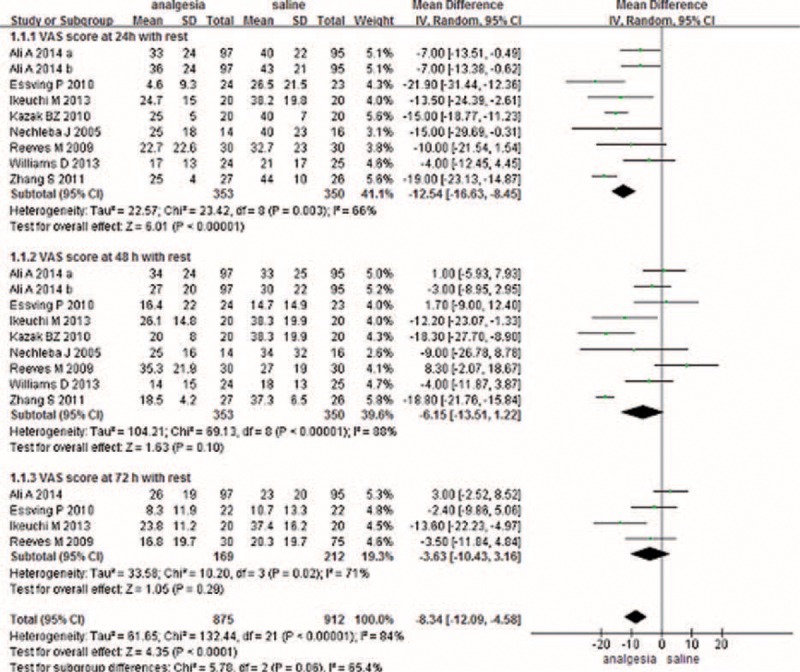
The meta-analysis of 8 trials included showed that CLIA show superiority than placebo in terms of VAS score with rest at 24 hours after TKA; however, there is no statistical significance between CLIA and placebo in terms of VAS score with rest at 48 and 72 hours after TKA. The alphabet “a” represents that the VAS score was measured at noon, and “b” means the VAS score was measured at 24 at pm. CLIA = continuous local infiltration analgesia, TKA = total knee arthroplasty, VAS = visual analog scale.

Only 8 studies with 703 patients showed the VAS score at 24 hours postoperatively with rest. Because 1 study reported the VAS score at 24 hours at different points, we also included the data in the meta-analysis; therefore, a total of 511 patients were included for the meta-analysis. The meta-analysis revealed that CLIA patients had a better outcome compared to the saline group with rest at 24 h in terms of their VAS score with rest at 24 h. (Figure 1) (MD −12.54, 95% CI −16.63 to −8.45, *P* < 0.000001).

A total of 8 component studies (903 patients) provided VAS scores at 48 hours postoperatively. There was no statistically significant difference between the groups with respect to the VAS score at 48 hours postoperatively (Figure 1) (MD −6.15, 95% CI −13.51 to 1.22, *P* = 0.10). There was statistical heterogeneity (*χ*^2^ = 46.39, df = 4, *I*^2^ = 88%, *P* < 0.00001), so a random-effects model was performed.

Four studies reported the VAS score with rest at 72 hours after surgery. There was no significant difference between the CLIA and saline group at 72 hours with rest (MD −3.63, 95% CI −10.43 to 3.16, *P* = 0.29) (Figure 1).

#### VAS Score with Mobilization

**FIGURE 2:**
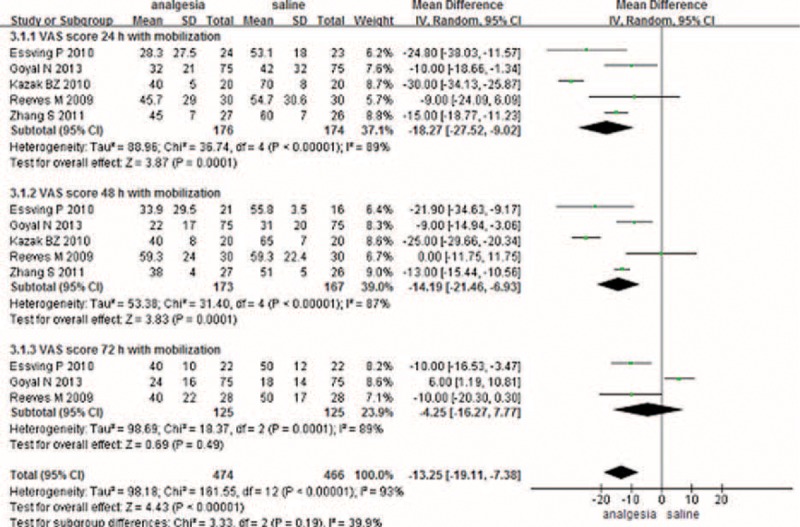
The meta-analysis of 5 trials included showed that CLIA show superiority than placebo in terms of VAS score with mobilization at 24 and 48 hours after TKA; however, there is no significant difference between the CLIA group and the placebo group in terms of VAS score with mobilization at 72 hours after TKA. CLIA = continuous local infiltration analgesia, TKA = total knee arthroplasty, VAS = visual analog scale.

A total of 5 component studies (350 patients) provided VAS scores at 24 hours with mobilization postoperatively. There was a statistically significant difference between the groups with respect to the VAS score at 24 hours postoperatively (Figure 2) (MD −18.27, 95% CI −18.27 to −9.02, *P* = 0.0001). Only 5 studies with 340 TKAs reported the VAS score at 48 hours postoperatively; meta-analysis found a significant difference between 2 groups (MD −14.19, 95% CI −21.46 to −6.93, *P* = 0.0001) (Figure 2). Three studies provided the VAS score with mobilization at 72 hours. The meta-analysis result showed that there was no significant difference between the CLIA and the placebo (MD −0.92, 95% CI −4.54 to 2.71, *P* = 0.62) (Figure 2).

#### Duration of Surgery

**FIGURE 3:**
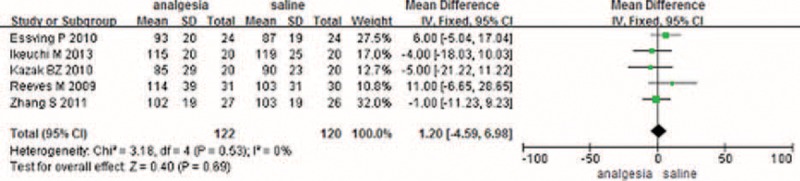
The meta-analysis of 5 trials included showed that CLIA shows no superiority than placebo in terms of surgery time after TKA. CLIA = continuous local infiltration analgesia, TKA = total knee arthroplasty.

A total of 5 studies addressed the duration of surgery between CLIA and placebo. There was no significant difference between the 2 groups (MD 1.20, 95% CI −4.59 to 6.98, *P* = 0.69) (Figure 3).

#### Length of Hospital Stay

**FIGURE 4:**
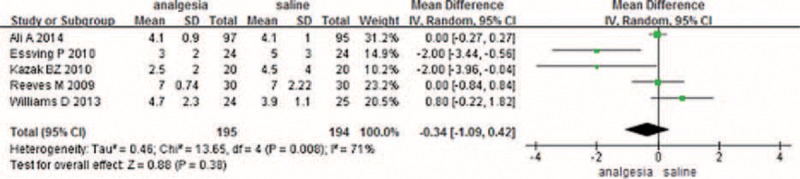
The meta-analysis of 3 trials included showed that CLIA shows no superiority than placebo in terms of length of hospital stay after TKA. CLIA = continuous local infiltration analgesia, TKA = total knee arthroplasty.

Five studies involving 398 patients reported the LOS between the 2 methods; the meta-analysis did not find a significant difference between the 2 methods (MD −0.34, 95% CI −1.09 to 0.42) (Figure 4).

#### Complications

**FIGURE 5:**
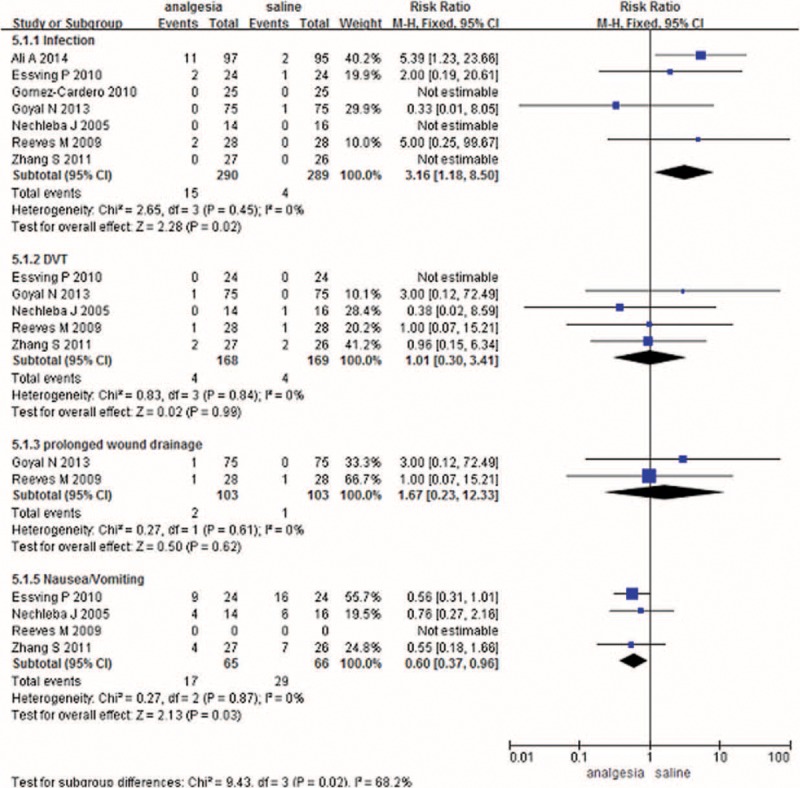
The meta-analysis of 6 trials included showed that there was no statistical significance between CLIA and placebo in terms of the rates of infection complication after TKA. CLIA = continuous local infiltration analgesia, TKA = total knee arthroplasty.

Seven studies paid close attention to postoperative infections. The meta-analysis reported a significant difference between the 2 methods in terms of postoperative infections (RR 3.61, 95% CI 1.18–8.50, *P* = 0.02) with no heterogeneity (Figure 5). Five studies investigated the occurrence of DVTs in both methods and found no significant difference between the 2 methods, suggesting that pain control after a TKA does not affect the incidence of DVT (RR 1.01, 95% CI 0.30–3.41, *P* = 0.99) (Figure 5). In addition to the above complications, 2 articles investigated whether CLIA can prolong wound drainage; there was no significant difference between CLIA and the control (RR 1.67, 95% CI 0.23–12.33, *P* = 0.62) (Figure 5). CLIA significantly reduced the incidences of nausea and vomiting after TKA (RR 0.60, 95% CI 0.37–0.96, *P* = 0.03) (Figure 5).

## DISCUSSION

To our knowledge, this is the first meta-analysis of RCTs comparing the efficacy and safety of CLIA with placebo in the management of pain after a TKA. The present meta-analysis was conducted on the basis of 10 randomized studies that found better pain control with rest at 24 hours and with mobilization at 24 and 48 hours postoperatively with CLIA compared with controls. There was no significant difference between the 2 groups with rest at 48 hours or with rest or mobilization at 72 hours. To our knowledge, pain control with mobilization is more important than pain control with rest, because good pain control with mobilization can facilitate early ambulation. The most important finding of this meta-analysis indicated that CLIA can increase the risk of infection, which is a concern of surgeons and will prolong the hospitalization time. In addition to the above-mentioned postoperative outcomes, other items were also analyzed, including the duration of surgery, the length of hospital stay and the incidence rates of nausea and vomiting, CLIA can decrease the occurrence rate of nausea of vomiting. One study was from 2005, and the rest of the studies were from 2009. All of the included studies were of high quality; only 2 studies did not use RCT methods, and 4 studies did not report their methodology. The double-blind method was used in all of the RCTs, and in only 1 study was the surgeon unaware of the pump's contents, which may impact the results of the present meta-analysis. All included studies showed comparable baseline data and provided the intention-to-treat analysis.

Local infiltration analgesia (LIA), as an effective method to reduce pain after TKA, has been used during TKA to reduce morphine consumption.^23,24^ The duration of LIA is only 24 hours; therefore, patients treated with LIA may experience more intense pain on the second day compared to the first postoperative day.^25^ To prolong the analgesic effect, continuous peripheral nerve block and continuous intra-articular analgesia have been attempted to control pain after TKA.^17,26^ The effect of continuous intra-articular infusion analgesia is still disputed because some articles have testified that it is an effective method to reduce pain intensity without increasing the chance of infection,^16,17,19^ whereas other studies have not drawn the same conclusion and have found a higher infection rate.^18,20^ As a result, a systematic review and meta-analysis are essential to identify the effect of CLIA in pain management after TKA.

Good pain management is especially important for TKA patients, as better pain control can lead to early mobilization, physiotherapy, and, most importantly, patient satisfaction.^5,27^ The results of our meta-analysis showed that CLIA led to better pain relief than the placebo with rest at 24 hours postoperative and with mobilization at 24 and 48 hours. However, there is no significant difference between rest at 48 or 72 hours and mobilization at 72 hours in terms of the VAS score. The reason for this can be easily interpreted because the short half-lives of bupivacaine (3.5 h) and ropivacaine (2–6 h) cannot provide sustained pain relief.^28^ As for the lack of efficacy at 48 hours with rest, this finding is supported by other studies examining postoperative knee replacement, anterior cruciate with rest at 48 hours, postop ligament reconstruction, and arthroscopic rotator cuff repair.^14,29–31^ From an overall perspective, CLIA may lead to better pain control after TKA and may thus increase patient satisfaction. Therefore, attempting to reduce the postoperative pain after TKA by using a continuous infusion of local anesthetic within 48 hours seems reasonable.

One of the greatest concerns of surgeons is the risk of infection postoperatively. Infection after a TKA is disastrous in terms of morbidity and cost because it will prolong the LOS. Our meta-analysis revealed that CLIA will increase the incidence of infection compared with the placebo group. Theoretically, the inflammatory mediators will be inhibited with CLIA,^16^ and local anesthetics have an antiseptic and fungistatic effect^32–34^; however, the reason behind this may be complex. On one hand, the pump preparation will increase the risk of infection because the analgesia group requires five 20-mL ampoules. On the other hand, the pump and catheter used for the CLIA will increase the risk of infection. Because the sample is not large enough, this outcome should treated cautiously. In our meta-analysis, the results show that CLIA will not prolong the LOS and thus will not have the potential to meaningfully increase the cost savings. As for the nausea and vomiting complications, the CLIA group had fewer incidences compared with the control group. Other complications, such as prolonged wound drainage, myocardial infarction, and DVT, showed no significant differences between the 2 groups.

The heterogeneity between the cited studies is primarily due to the mixture of different analgesics and different dosages, which may impact our findings regarding the CLIA method. There are 2 primary methods of performing CLIA: one involves a pump, and the other involves an intra-articular bolus injection. We could not determine the optimal concentration and methods to obtain the best analgesic effect; however, CLIA can offer pain relief at 24 and 48 hours with mobilization. As for the complication of infection, 4 studies reported that an infection occurred after TKA. However, only 1 study^12^ reported a significant difference between the 2 groups, and that study did not provide any rationale as to why there was a difference.

There were several limitations in this meta-analysis: only 10 RCTs were included, and the sample sizes in each trial were small, which would affect the final results; the duration of follow-up in some studies was unclear, and long-term follow-up was needed for this analysis; the publication bias that existed in the meta-analysis also influenced the results; and the CLIA method and the pump transfusion rate and volume were all different, which also likely had an effect on the final results.

## CONCLUSIONS

In conclusion, the results of our meta-analysis indicate that CLIA may be more effective at 24 hours with rest and at 24 and 48 hours with mobilization compared with the placebo. The most important finding of this study is that CLIA may also increase the rate of infection. Moreover, there is no significant difference in CLIA or placebo treatment for pain at 48 hours with rest and at 72 hours with rest or mobilization. For future research, optimal drugs and drug dosages should be rigorously defined, and the method of CLIA should also be clarified. Even more importantly, well designed trials with larger sample sizes are needed to further provide reliable evidence on the safety of CLIA for pain management after a TKA. This was the first systematic review to evaluate the efficacy and safety of CLIA compared with placebo on reducing pain after a TKA. High-quality RCTs and well designed trials are still required to detect differences in knee flexion and other outcomes after a TKA.
